# Leptin Receptors in RIP-Cre^25Mgn^ Neurons Mediate Anti-dyslipidemia Effects of Leptin in Insulin-Deficient Mice

**DOI:** 10.3389/fendo.2020.588447

**Published:** 2020-09-23

**Authors:** Ashish Singha, Juan Pablo Palavicini, Meixia Pan, Scotlynn Farmer, Darleen Sandoval, Xianlin Han, Teppei Fujikawa

**Affiliations:** ^1^Department of Cellular and Integrative Physiology, Long School of Medicine, University of Texas Health San Antonio, San Antonio, TX, United States; ^2^Barshop Institute for Longevity and Aging Studies, University of Texas Health San Antonio, San Antonio, TX, United States; ^3^Department of Surgery, University of Michigan, Ann Arbor, MI, United States; ^4^Center for Biomedical Neuroscience, University of Texas Health San Antonio, San Antonio, TX, United States; ^5^Division of Hypothalamic Research Center, Internal Medicine, UT Southwestern Medical Center at Dallas, Dallas, TX, United States

**Keywords:** leptin, insulin deficiency, the hypothalamus, glucose metabolism, lipid metabolism

## Abstract

Leptin is a potent endocrine hormone produced by adipose tissue and regulates a broad range of whole-body metabolism such as glucose and lipid metabolism, even without insulin. Central leptin signaling can lower hyperglycemia in insulin-deficient rodents via multiple mechanisms, including improvements of dyslipidemia. However, the specific neurons that regulate anti-dyslipidemia effects of leptin remain unidentified. Here we report that leptin receptors (LEPRs) in neurons expressing Cre recombinase driven by a short fragment of a promoter region of *Ins2* gene (RIP-Cre^25Mgn^ neurons) are required for central leptin signaling to reverse dyslipidemia, thereby hyperglycemia in insulin-deficient mice. Ablation of LEPRs in RIP-Cre^25Mgn^ neurons completely blocks glucose-lowering effects of leptin in insulin-deficient mice. Further investigations reveal that insulin-deficient mice lacking LEPRs in RIP-Cre^25Mgn^ neurons (RIP-Cre^ΔLEPR^ mice) exhibit greater lipid levels in blood and liver compared to wild-type controls, and that leptin injection into the brain does not suppress dyslipidemia in insulin-deficient RIP-Cre^ΔLEPR^ mice. Leptin administration into the brain combined with acipimox, which lowers blood lipids by suppressing triglyceride lipase activity, can restore normal glycemia in insulin-deficient RIP-Cre^ΔLEPR^ mice, suggesting that excess circulating lipids are a driving-force of hyperglycemia in these mice. Collectively, our data demonstrate that LEPRs in RIP-Cre^25Mgn^ neurons significantly contribute to glucose-lowering effects of leptin in an insulin-independent manner by improving dyslipidemia.

## Introduction

Central leptin injections can maintain euglycemic ranges in insulin-deficient rodent models without exogenous insulin administration (1–6). Previous studies have unraveled key neuronal components contributing to glucose-lowering effects of central leptin signaling (3, 4, 6–8). Among these identified groups, leptin receptors (LEPRs) in GABAergic neurons substantially contribute to glucose-lowering effects of leptin in an insulin-independent manner (6). Intriguingly, leptin-responsive GABAergic neurons are restrictedly positioned to the hypothalamic arcuate nucleus (ARC), dorsomedial nucleus (DMH), and lateral areas (LHA) (6, 9). Of note, among these three areas, the vast of majority of leptin-responsive GABAergic neurons are located in the ARC and DMH (9). Recent studies have further shown that LEPRs in agouti-related peptide-expressing neurons (AgRP neurons), which are GABAergic and located in the ARC, are key to glucose-lowering effects of central leptin signaling (7, 8). However, other GABAergic neuronal groups likely contribute to glucose-lowering effects as well, because intracerebroventricular (i.c.v.) leptin injection still can lower hyperglycemia in insulin-deficient mice lacking LEPRs in AgRP neurons (8). Identification of neuronal groups underlying glucose-lowering effects of leptin in an insulin-independent manner has not yet been achieved.

A study using single cell RNA-sequence shows that GABAergic neurons in the ARC and median eminence (Arc-ME) complex are composed of distinct genetically-defined neuronal groups (10). AgRP neurons are the most dominant neurons among Arc-ME GABAergic neurons (10). In the same study, it reveals that neurons expressing Cre recombinase driven by a short fragment of rat insulin promoter transgene (RIP-Cre^25Mgn^ neurons) are distinguished from AgRP neurons, and uniquely composed from several neuronal groups (10). A RIP-Cre^25Mgn^ mouse line was originally generated to target pancreatic β-cells (11). However, the mice ectopically express Cre recombinase in the central nervous system (CNS) (12, 13) due to the nature of genetically-engineering methods in the early era (14, 15). Because RIP-Cre^25Mgn^ mice express Cre recombinase in unique and distinct neurons from conventional hypothalamic neurons such as AgRP neurons (10, 16), the transgenic mice have been utilized for studies investigating the role of unconventional hypothalamic neurons, in particular focusing on the regulation of metabolism (17–20). RIP-Cre^25Mgn^ neurons regulate energy expenditure (17, 20) and glucose and fat metabolism (18, 19) in the presence of insulin. Mice lacking LEPRs in RIP-Cre^25Mgn^ cells show aberrant fat metabolism, including modest increases in body weight, insulin, and triglyceride (TG) (21). Because LEPRs are not express in pancreatic β-cells (6, 22), metabolic phenotypes in mice lacking LEPRs in RIP-Cre^25Mgn^ cells result from ablation of LEPRs in the CNS.

The anatomical profiling of GABAergic RIP-Cre^25Mgn^ neurons is very similar to that of leptin-responsive GABAergic neurons (6, 9). GABAergic RIP-Cre^25Mgn^ neurons are located in restricted areas, the ARC, DMH, and the medial tuberal nucleus (MTu) (17). Of note, GABAergic RIP-Cre^25Mgn^ neurons only represent a portion of leptin-responsive GABAergic neurons because the phenotypic differences between mice lacking LEPRs in GABAergic neurons (GABA^ΔLEPR^) and RIP-Cre^25Mgn^ neurons (RIP-Cre^ΔLEPR^) are enormous, for instance, body weight of GABA^ΔLEPR^ mice is near identical to that of *db/db* mice (9) while RIP-Cre^ΔLEPR^ mice only show modest increases in body weight (21). Based on the metabolic roles in glucose and lipid metabolism and anatomical profile of RIP-Cre^25Mgn^ neurons, we reasoned that LEPRs in RIP-Cre^25Mgn^ neurons significantly contribute to glucose-lowering effects of leptin in an insulin-independent manner. To test our hypothesis, we generated insulin-deficient mice lacking LEPRs in RIP-Cre^25Mgn^ neurons (RIP-Cre^ΔLEPR^) and examined whether i.c.v. leptin injection can lower hyperglycemia in these mice without insulin. We found that deletion of LEPRs in RIP-Cre^25Mgn^ neurons blocks glucose- and lipid-lowering effects of leptin in insulin-deficient mice. Further, we found that administration of lipid-lowering compound acipimox can restore glucose-lowering effects of leptin in insulin-deficient RIP-Cre^ΔLEPR^ mice. Our results indicate that RIP-Cre^25Mgn^ neurons are vital components for glucose-lowering effects of leptin through the regulation of lipid metabolism in an insulin-independent manner.

## Materials and Methods

### Genetically-Engineered Mice

RIP-Cre^25Mgn^ (11) and Ai9 mice (23) were obtained from the Jackson Laboratory (JAX, USA, #003573 and #007909). *Lepr*^flox/−^ (24) and *Lepr*^loxTB/WT^ (25) mice were obtained from Dr. Joel Elmquist at the University of Texas Southwestern Medical Center (UTSW), and are also available at the JAX (#008327 and #018989). RIP^Herr^-DTR mice (26) were obtained from Dr. Pedro Herrera at Geneva University. *Gcg*^loxTB/WT^ mice were generated as previously described (27). To generate mice lacking LEPRs in RIP-Cre^25Mgn^ neurons, we bred RIP-Cre^25Mgn^ with *Lepr*^flox/−^ mice followed by breeding RIP-Cre^25Mgn^::*Lepr*^flox/flox^ with *Lepr*^flox/flox^. To generate mice re-expressing LEPRs in RIP-Cre^25Mgn^ neurons otherwise LEPRs null, we bred RIP-Cre^25Mgn^ with *Lepr*^loxTB/WT^ mice followed by breeding RIP-Cre^25Mgn^::*Lepr*^loxTB/WT^ with *Lepr*^loxTB/WT^. To genetically induce insulin deficiency, RIP^Herr^-DTR mice (26) were bred with mice described above and inject diphtheria toxin. To examine effects of diphtheria toxin injections on the viability of RIP-Cre^25Mgn^ neurons, we introduced the tdTomato allele (23) to identify RIP-Cre^25Mgn^ neurons under the fluorescent microscopy. Mice used are as follows; RIP-Cre^25Mgn^::*Lepr*^flox/flox^::RIP^Herr^-DTR, *Lepr*^flox/flox^::RIP^Herr^-DTR (control for RIP-Cre^25Mgn^::*Lepr*^flox/flox^::RIP^Herr^-DTR), RIP-Cre^25Mgn^::*Lepr*^loxTB/loxTB^::RIP^Herr^-DTR*, Lepr*^loxTB/loxTB^::RIP^Herr^-DTR (null control for RIP-Cre^25Mgn^::*Lepr*^loxTB/loxTB^::RIP^Herr^-DTR), *Lepr*^WT/WT^::RIP^Herr^-DTR and RIP-Cre^25Mgn^::*Lepr*^WT/WT^::RIP^Herr^-DTR (wild-type control for RIP-Cre^25Mgn^::*Lepr*^loxTB/loxTB^::RIP^Herr^-DTR), *Gcg*^loxTB/loxTB^::RIP^Herr^-DTR, *Gcg*^WT/WT^::RIP^Herr^-DTR (control for *Gcg*^loxTB/loxTB^ ::RIP^Herr^-DTR), and RIP-Cre^25Mgn^::RIP^Herr^-DTR::Ai9^TB/−^. We used KAPA Mouse genotyping kits (KAPA Biosystems, USA) to determine genotypes. All genotyping primers and predicted band sizes are described in Supplementary Table 1. We used 3–6 month-old male mice whose body weights were above ~25 grams. All mice were fed with a normal chow diet (Teklad LM-485, Envigo, US). Animal care was according to established NIH guidelines, and all procedures were approved by the Institutional Animal Care and Use Committee of the University of Texas Southwestern Medical Center and University of Texas Health San Antonio.

### Assessment of Basal Metabolism Prior to Induction of Insulin Deficiency

We measured body weight weekly after weaning at 8 weeks of age. Blood glucose and plasma insulin were measured at 8–12 weeks of ages prior to inducing insulin deficiency. Body composition of RIP-Cre^25Mgn^::*Lepr*^loxTB/loxTB^::RIP^Herr^-DTR was measured by rodent fMRI as previously described (1, 28, 29). Blood glucose was measured with a commercially available glucose monitor (Bayer Contour, USA). Insulin was measured using a commercially available ELISA kit (Crystal Chem, USA) (30).

### Induction of Insulin Deficiency by a RIP-DTR Approach

To induce insulin deficiency, mice were treated with diphtheria toxin (DT, Sigma, USA). DT was dissolved in sterile 0.9% NaCl solution at a concentration of 150 μg/mL and kept at −80°C until use. Each concentrated DT aliquot was diluted to 0.075 μg/mL in sterile saline and delivered intraperitoneally (i.p) at a dose of 0.5 μg/kg B.W. one time per day for 3 consecutive days to ablate pancreatic β-cells (**Figure 2A**). As previously described (1, 6, 8, 26), blood insulin levels were not detectable by the ELISA kit in all mice models (Sensitively threshold was 50 pg/mL) except for RIP-Cre^25Mgn^::*Lepr*^loxTB/loxTB^::RIP^Herr^-DTR and *Lepr*^loxTB/loxTB^::RIP^Herr^-DTR after DT injections (Supplementary Figure 1 and **Figure 3H**).

### Leptin Administration Into the Brain

Leptin (Peprotech, USA; 25 ng/h/0.11 μL) was dissolved in sterile phosphate-buffered saline (PBS; pH = 7.4, Invitrogen, US) and administered by intracerebroventricular (i.c.v.) infusion using osmotic pumps (Alzet, US) as previously described (1, 6, 8). An osmotic minipump designed for use in mice (model 1004; Alzet) was implanted subcutaneously and attached via a catheter to the lateral ventricle cannula for i.c.v. administration. PBS was administered to the control group as a placebo treatment. We continuously administered leptin for up to 25 days as pumps are designed to deliver for ~28 days.

### Measurement of Metabolic Parameters and Survival

We measured glucose, body weight, and food intake every 5 days as previously described (1, 6, 8). We plotted survival to determine if LEPRs in RIP-Cre^25Mgn^ neurons are required or sufficient for leptin's capacity to reduce lethality in insulin-deficient mice. Free fatty acids (FFAs), ketone bodies, TG, and glycerol in blood were measured by commercially available kits (Wako diagnose, US; and Cayman US for glycerol) as described previously (1). Corticosterone (Cayman, US) and glucagon (#10-1271-01, Mercodia, US) in the plasma were measured by commercially available ELISA kits.

### Immunohistochemistry

Mice were deeply anesthetized with isoflurane and underwent transcardiac perfusion fixation with 4% paraformaldehyde as previously described (31). After cryoprotection in 30% sucrose-sterile-PBS solution, the brain was cut in 25 μm sections using a freezing microtome. Brain sections were mounted on glass slides using the antifade mounting medium with DAPI (H-1500, Vector Lab, USA). Images were captured by fluorescence microscopy (Keyence US, US; Model: BZ-X710). Neurons expressing tdTomato fluorescent and distributed in the hypothalamus at the coronal section ~-0.5 to −2.0 mm from the caudal to the bregma were manually counted.

### Assessment of mRNA

Mice were deeply anesthetized with isoflurane and tissues were quickly removed, frozen in liquid nitrogen and subsequently stored at −80°C. RNA was extracted using STAT60 reagent (Amsbio, MA, USA). Complementary DNA from 1 μg of input RNA was generated with the High Capacity cDNA Reverse Transcription Kits (Life Technologies). SYBR Green PCR master mix (Life Technologies) was used for the quantitative real time PCR analysis. Sequences of deoxy-oligonucleotides primers are outlined in Supplementary Table 2.

### Assessment of Hepatic Lipids

Liver tissue was homogenized in ice-cold diluted phosphate-buffered saline (0.1X PBS) as described previously (32). Lipids were extracted by a modified procedure of Bligh and Dyer extraction in the presence of internal standards which were added based on the total protein content of individual samples as described previously (33–35). A triple-quadrupole mass spectrometer (Thermo Scientific TSQ Altis, CA, USA) and a Quadrupole-Orbitrap™ mass spectrometer (Thermo Q Exactive™, San Jose, CA) equipped with a Nanomate device (Advion Bioscience Ltd., NY, USA) and Xcalibur system software was used as previously described (36–38). Briefly, diluted lipid extracts were directly infused into the ESI source through a Nanomate device. Typically, signals were averaged over a 1-min period in the profile mode for each full scan MS spectrum. For tandem MS, a collision gas pressure was set at 1.0 mTorr, but the collision energy varied with the classes of lipids. Similarly, a 2–5-min period of signal averaging in the profile mode was employed for each tandem MS mass spectrum. All full and tandem MS mass spectra were automatically acquired using a customized sequence subroutine operated under Xcalibur software. Data processing including ion peak selection, baseline correction, data transfer, peak intensity comparison, ^13^C deisotoping, and quantitation were conducted using a custom programmed Microsoft Excel macro as previously described after considering the principles of lipidomics (37, 38).

### Injection of Acipimox

Acipimox (Sigma Aldrich, US) was i.p. administered at a dose of 100 mg/kg B.W. two times per day for five consecutive days. Control solution was sterile saline (0.9% NaCl).

### Data Analysis

Data are represented as the group mean ± S.E.M. as indicated in each figure legend. Statistical significance was determined using GraphPad PRISM software (ver8, GraphPad, San Diego, CA) by unpaired *t*-test, one-way ANOVA followed by Turkey's multiple comparison test, two-way ANOVA followed by one-way ANOVA (Tukey's multiple comparison test if the interaction was significant) or unpaired *t*-test in the same factor (if the interaction was not significant), or repeated measures ANOVA followed by unpaired *t*-test if the interaction was significant. For analysis of survival curves, Log-rank (Mantel-Cox) testing was used. Since the number of mice surviving declined over time, we were prohibited from utilizing repeated measures ANOVA (**Figures 2B–G**, and **Figures 4D–H**). We, therefore, performed the statistical analysis and showed each day individually in **Figures 2C–H** and **Figure 4E**. For all tests, statistical significance was set at a critical value of *P* < 0.05.

## Results

### Validation of Insulin-Deficient RIP-Cre^ΔLEPR^ Mice

**Figure 1:**
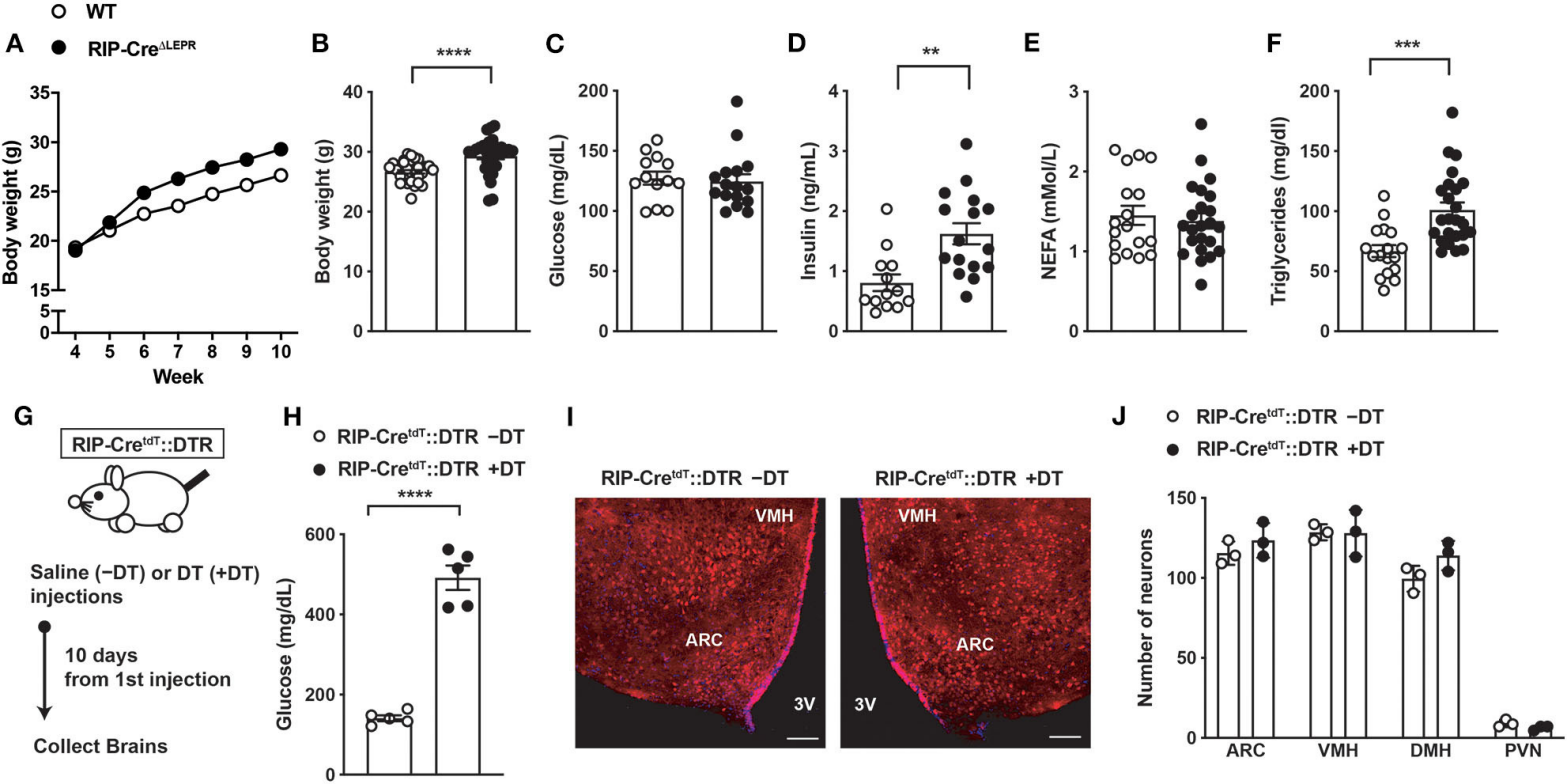
Induction of insulin deficiency with a RIP-DTR approach does not affect RIP-Cre^25Mgn^ neurons. **(A)** The time course of body weight (*n* = 33; WT and 30; RIP-Cre^ΔLEPR^), **(B)** body weight (*n* = 33; WT and 30; RIP-Cre^ΔLEPR^), **(C)** blood glucose (*n* = 13; WT and 16; RIP-Cre^ΔLEPR^), **(D)** plasma insulin (*n* = 13; WT and 16; RIP-Cre^ΔLEPR^), **(E)** plasma NEFA (*n* = 17; WT and 23; RIP-Cre^ΔLEPR^), **(F)** plasma TG (*n* = 17; WT and 24; RIP-Cre^ΔLEPR^) in mice lacking LEPRs in RIP-Cre^25Mgn^ neurons (RIP-Cre^ΔLEPR^) at 10 weeks of ages. Wild type control (WT) were homozygous *Lepr*^flox^ without RIP-Cre^25Mgn^ transgene. **(G)** A schematic design for experiments for H, I, and J. Mice were i.p., administered with DT (3 times [0, 1, 2 days], 0.5 μg/kg B.W.) **(H)** Blood glucose levels (*n* = 5; RIP-Cre^ΔLEPR^ –DT and 5; RIP-Cre^ΔLEPR^ +DT), **(I)** representative figures (left; Saline injection, right; DT injections, white bar represents 100 μm) and **(J)** the number of tdTomato positive cells in the hypothalamus 10 days after the induction of insulin deficiency in RIP-Cre^ΔLEPR^ mice expressing tdTomato in RIP-Cre^25Mgn^ neurons by a RIP-DTR approach (*n* = 3; RIP-Cre^ΔLEPR^ –DT and 3; RIP-Cre^ΔLEPR^ +DT). VMH, the ventromedial hypothalamic nucleus; ARC, the hypothalamic arcuate nucleus, 3V, third ventricular; DMH, dorsomedial hypothalamic nucleus; PVN, the paraventricular hypothalamic nucleus. Values are mean ± S.E.M. ****p < 0.0001, ***p < 0.001, **p <0.01. Unpaired *t*-test was used to analyze the data.

In agreement with a previous report (21), in the presence of insulin, we found that RIP-Cre^ΔLEPR^ mice did not show significant differences of blood glucose and FFAs, while they exhibited modest increases in body weight and higher circulating insulin and TG levels compared to WT group (Figures 1A–F). To induce insulin deficiency in RIP-Cre^ΔLEPR^ mice, we utilize a RIP^Herr^-DTR approach (26). After DT injections, RIP-Cre^ΔLEPR^ mice exhibited hyperglycemia and insulin deficiency (Supplementary Figures 1B,C). Because the study shows that a short fragment of RIP^Herr^ promoter drives Cre-recombinase in the hypothalamus (12), we previously determined whether a RIP^Herr^-DTR approach affects hypothalamic neurons (6, 8). DT injections into RIP^Herr^-DTR mice did not reduce the number of AgRP and proopiomelanocortin (POMC) neurons, and mRNA levels of *Pomc, Agrp, and Ins2* in the mediobasal hypothalamus (6, 8). These data suggest that a RIP^Herr^-DTR approach do not ablate hypothalamic neurons, most likely because (i) a RIP^Herr^ fragment transgene drives genes in lesser ectopic expression levels than other RIP fragments do (12), and (ii) the dose of DT we used is ~100 times less compared to studies aiming to ablate DTR-expressing neurons (39, 40). Nonetheless, we examined if DT injections ablate RIP-Cre^25Mgn^ neurons. To do so, we administered DT (3 times, one injection per day, 0.5 μg per kg B.W.) into RIP-Cre^25Mgn^::RIP^Herr^-DTR::*tdTomato*^TB/TB^ mice, which allow us to visualize RIP-Cre^25Mgn^ neurons by a red fluorescent reporter tdTomato (Figure 1G). Ten days after the first injection of DT that was sufficient to induce hyperglycemia and insulin deficiency (Figure 1H and Supplementary Figure 1), we examined if DT injections would ablate RIP-Cre^25Mgn^ neurons. We did not find any significant differences in numbers of tdTomato positive cells in the hypothalamus after DT injections (Figures 1I,J), confirming that our RIP^Herr^-DTR approach does not ablate hypothalamic RIP-Cre^25Mgn^ neurons, similar to AgRP (8), POMC, and hypothalamic GABAergic neurons (6).

### LEPRs in RIP-Cre^25Mgn^ Neurons Are Required, but Not Sufficient for Glucose-Lowering Effects of Leptin

**Figure 2:**
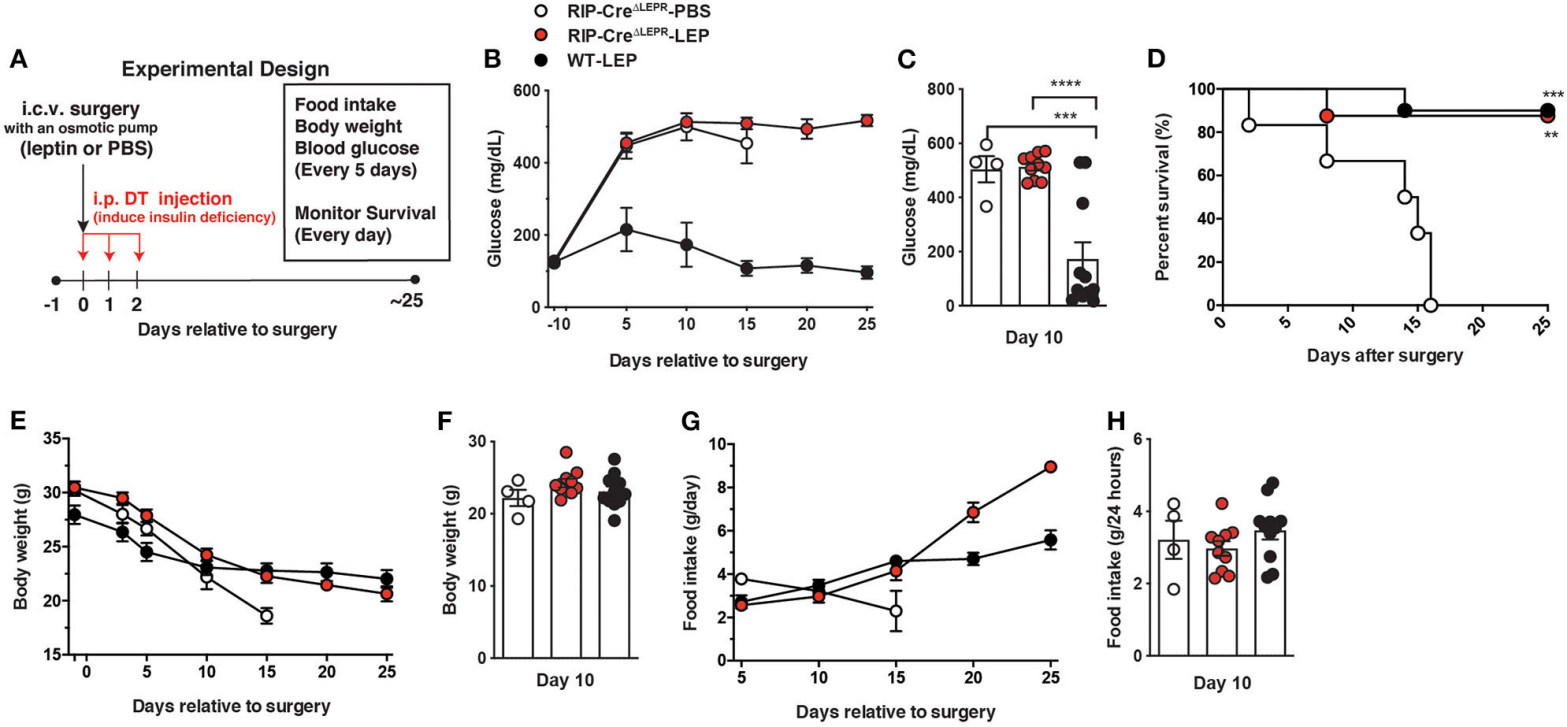
Deletion of LEPRs in RIP-Cre^25Mgn^ neurons blocks glucose-lowering effects of leptin in insulin-deficient mice. **(A)** Experimental design for i.c.v. leptin injection into insulin-deficient mice. **(B)** The time course of blood glucose (*n* = 5; RIP-Cre^ΔLEPR^-PBS, 11; RIP-Cre^ΔLEPR^-LEP, and 11; WT-LEP at day −1), **(C)** blood glucose at Days 10 (*n* = 4; RIP-Cre^ΔLEPR^-PBS, 10; RIP-Cre^ΔLEPR^-LEP, and 11; WT-LEP at day −1), **(D)** the time course of survival percentage (*n* = 5; RIP-Cre^ΔLEPR^-PBS, 11; RIP-Cre^ΔLEPR^-LEP, and 11; WT-LEP at day 0), **(E)** the time course of body weight (*n* = 4; RIP-Cre^ΔLEPR^-PBS, 11; RIP-Cre^ΔLEPR^-LEP, and 11; WT-LEP at day −1), **(F)** body weight at Days 10 (*n* = 4; RIP-Cre^ΔLEPR^-PBS, 10; RIP-Cre^ΔLEPR^-LEP, and 11; WT-LEP at day −1), **(G)** the time course of food intake 10 (*n* = 4; RIP-Cre^ΔLEPR^-PBS, 10; RIP-Cre^ΔLEPR^-LEP, and 11; WT-LEP at day 5), and **(H)** food intake at Days 10 (*n* = 4; RIP-Cre^ΔLEPR^-PBS, 10; RIP-Cre^ΔLEPR^-LEP, and 11; WT-LEP at day −1) in insulin-deficient RIP-Cre^ΔLEPR^ mice chronically administered leptin into the lateral ventricle (25 ng/0.11 μL/h). Insulin deficiency was induced by administration of DT. Control group for leptin administration was administered sterile vehicle (PBS). Genetic control group (WT) were homozygous *Lepr*^flox^ without RIP-Cre^25Mgn^ transgene. Values are mean ± S.E.M. ****p < 0.0001, ***p < 0.001, **p <0.01. One way ANOVA followed by Tukey's multiple comparison test **(C,F,H)** and Log-rank (Mantel-Cox) testing **(D)** was used to analyze the data.

Next, we asked if LEPRs in RIP-Cre^25Mgn^ neurons are required for glucose-lowering effects in an insulin-independent manner. To this end, we administered leptin into the lateral ventricular of insulin-deficient RIP-Cre^ΔLEPR^ mice and examined blood glucose levels. The experimental design is illustrated in Figure 2A. Intriguingly, chronic i.c.v. leptin injection did not reverse hyperglycemia in insulin-deficient RIP-Cre^ΔLEPR^ mice (Figures 2B,C), suggesting that LEPRs in RIP-Cre^25Mgn^ neurons play a critical role in glucose-lowering effects of leptin in an insulin-independent manner. As we expected, insulin deficiency decreased survival of RIP-Cre^ΔLEPR^ mice administered PBS (RIP-Cre^ΔLEPR^-PBS; Of note, from here, all abbreviations of groups containing either -PBS or -LEP are insulin-deficient mice) within 2–3 weeks after the induction of insulin deficiency (Figure 2D). Surprisingly, the survival rate of insulin-deficient RIP-Cre^ΔLEPR^ mice administered leptin (RIP-Cre^ΔLEPR^-LEP) was comparable to insulin-deficient WT mice administered leptin (WT-LEP) (Figure 2D) despite hyperglycemia. Previously, our studies have shown that there is no correlation between the improvement of blood glucose and survival probability after i.c.v. leptin injection (1, 6, 8), and this study further confirmed this notion. Body weight between RIP-Cre^ΔLEPR^-LEP and WT-LEP was comparable 10 days after leptin administration was initiated (Figures 2E,F). Food intake was comparable until 15 days after leptin administration was initiated (Figures 2G,H). Previous studies clearly have shown that the amount of food intake could not explain glucose-lowering effects of leptin (1, 41). At Day 10, RIP-Cre^ΔLEPR^-LEP showed significantly higher blood glucose compared to WT-LEP, yet the amount of food intake between RIP-Cre^ΔLEPR^-LEP and WT-LEP was comparable (Figures 2C,H). These data demonstrated that it is unlikely that hyperglycemia in RIP-Cre^ΔLEPR^-LEP resulted from the differences of food intake or body weight after induction of insulin-deficiency. However, we cannot exclude the possibility that the increased food intake 20 days after induction of insulin-deficiency contributed to some of the hyperglycemia observed at that time in RIP-Cre^ΔLEPR^-LEP vs. WT-LEP (Figure 2G).

**Figure 3:**
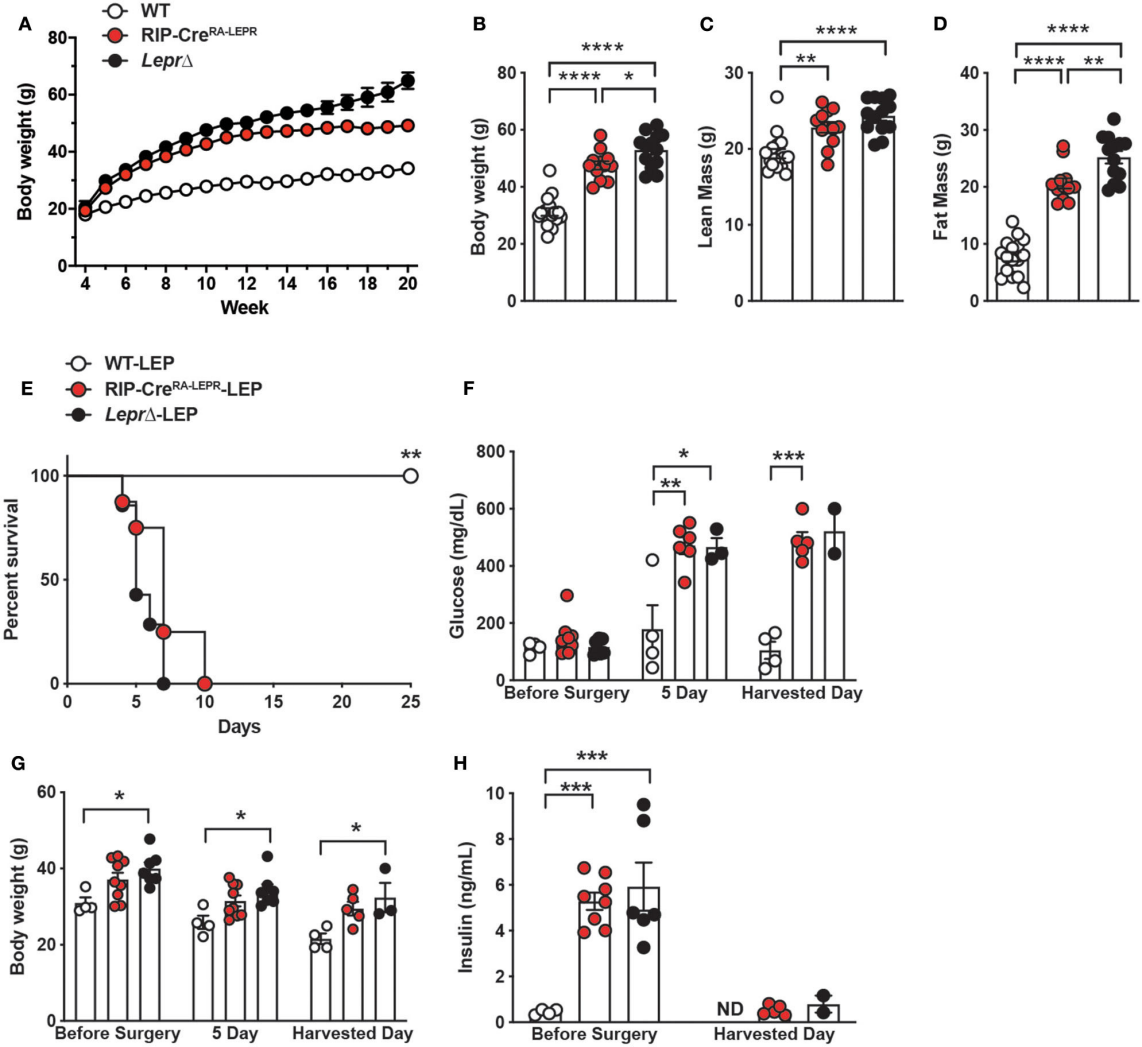
Re-expression of LEPRs in RIP-Cre^25Mgn^ neurons is not sufficient for leptin to lower glucose in insulin-deficient mice. **(A)** The time course of body weight (*n* = 16; WT, 12; RIP-Cre^RA−LEPR^, and 13; *Lepr*Δ), **(B)** body weight (*n* = 16; WT, 12; RIP-Cre^RA−LEPR^, and 13; *Lepr*Δ), **(C)** lean mass (*n* = 16; WT, 12; RIP-Cre^RA−LEPR^, and 13; *Lepr*Δ), and **(D)** fat mass at 15–16 (*n* = 16; WT, 12; RIP-Cre^RA−LEPR^, and 13; *Lepr*Δ) weeks of ages of mice re-expressing LEPRs only in RIP-Cre^25Mgn^ neurons (RIP-Cre^RA−LEPR^). Wild-type control (WT) was composed of RIP-Cre^25Mgn^::*Lepr*^WT/WT^ and *Lepr*^WT/WT^, and *Lepr*^loxTB/loxTB^ mice were used as LEPRs-deficient mice. **(E)** The survival percentage (*n* = 4; WT-LEP, 9; RIP-Cre^RA−LEPR^-LEP, and 7; *Lepr*Δ-LEP at day −1), **(F)** blood glucose (*n* = 4; WT-LEP, 9; RIP-Cre^RA−LEPR^-LEP, and 7; *Lepr*Δ-LEP at day −1, *n* = 4; WT-LEP, 5; RIP-Cre^RA−LEPR^-LEP, and 3; *Lepr*Δ-LEP at day 5, *n* = 4; WT-LEP, 4; RIP-Cre^RA−LEPR^-LEP, and 2; *Lepr*Δ-LEP at the harvest day), **(G)** body weight (*n* = 4; WT-LEP, 9; RIP-Cre^RA−LEPR^-LEP, and 7; *Lepr*Δ-LEP at day −1, *n* = 4; WT-LEP, 5; RIP-Cre^RA−LEPR^-LEP, and 3; *Lepr*Δ-LEP at day 5, *n* = 4; WT-LEP, 4; RIP-Cre^RA−LEPR^-LEP, and 2; *Lepr*Δ-LEP at the harvest day), and **(H)** plasma insulin levels (*n* = 4; WT-LEP, 9; RIP-Cre^RA−LEPR^-LEP, and 7; *Lepr*Δ-LEP at day −1, *n* = 4; WT-LEP, 5; RIP-Cre^RA−LEPR^-LEP, and 3; *Lepr*Δ-LEP at day 5, *n* = 4; WT-LEP, 4; RIP-Cre^RA−LEPR^-LEP, and 2; *Lepr*Δ-LEP at the harvest day) in insulin-deficient RIP-Cre^RA−LEPR^ mice chronically administered leptin into the lateral ventricle (25 ng/0.11 μL/h). Harvested day means the date of death of succumbed RIP-Cre^RA−LEPR^-LEP and *Lepr*Δ-LEP mice and 25 days for WT-LEP. Values are mean ± S.E.M. ****p < 0.0001, ***p < 0.001, **p <0.01, *p < 0.05. One way ANOVA followed by Tukey's multiple comparison test **(B–D,F–H)** and Log-rank (Mantel-Cox) testing **(E)** was used to analyze the data.

We further asked if expression of LEPRs only in RIP-Cre^25Mgn^ neurons is sufficient for leptin to exert its glucose-lowering effects. To do so, we generated mice re-expressing LEPRs in RIP-Cre^25Mgn^ neurons (RIP-Cre^RA−LEPR^). RIP-Cre^RA−LEPR^ mice had a similar body weight up to 14–15 weeks of ages compared to LEPRs null mice (*Lepr*Δ) (Figure 3A). Starting at 15 weeks of age, RIP-Cre^RA−LEPR^ mice had significantly lower body weight along with reduced fat mass compared to *Lepr*Δ mice (Figures 3B,D). However, the body weight of RIP-Cre^RA−LEPR^ mice was still extremely higher than WT control mice (Figures 3A–D). We chronically administered leptin into the lateral ventricular of DT-injected RIP-Cre^RA−LEPR^ mice (RIP-Cre^RA−LEPR^-LEP). We did not see any improvements of the survival rate, blood glucose, body weight of RIP-Cre^RA−LEPR^-LEP compared to *Lepr*Δ-LEP (Figures 2E–H), although these groups still showed a tiny residue of insulin in blood after DT injections. Collectively, these data indicate that LEPRs in RIP-Cre^25Mgn^ neurons are required, but not sufficient for the insulin-independent glucose-lowering effects of leptin.

### Glucose-Lowering Effects of Leptin Are Independent of Glucagon Singling

**Figure 4:**
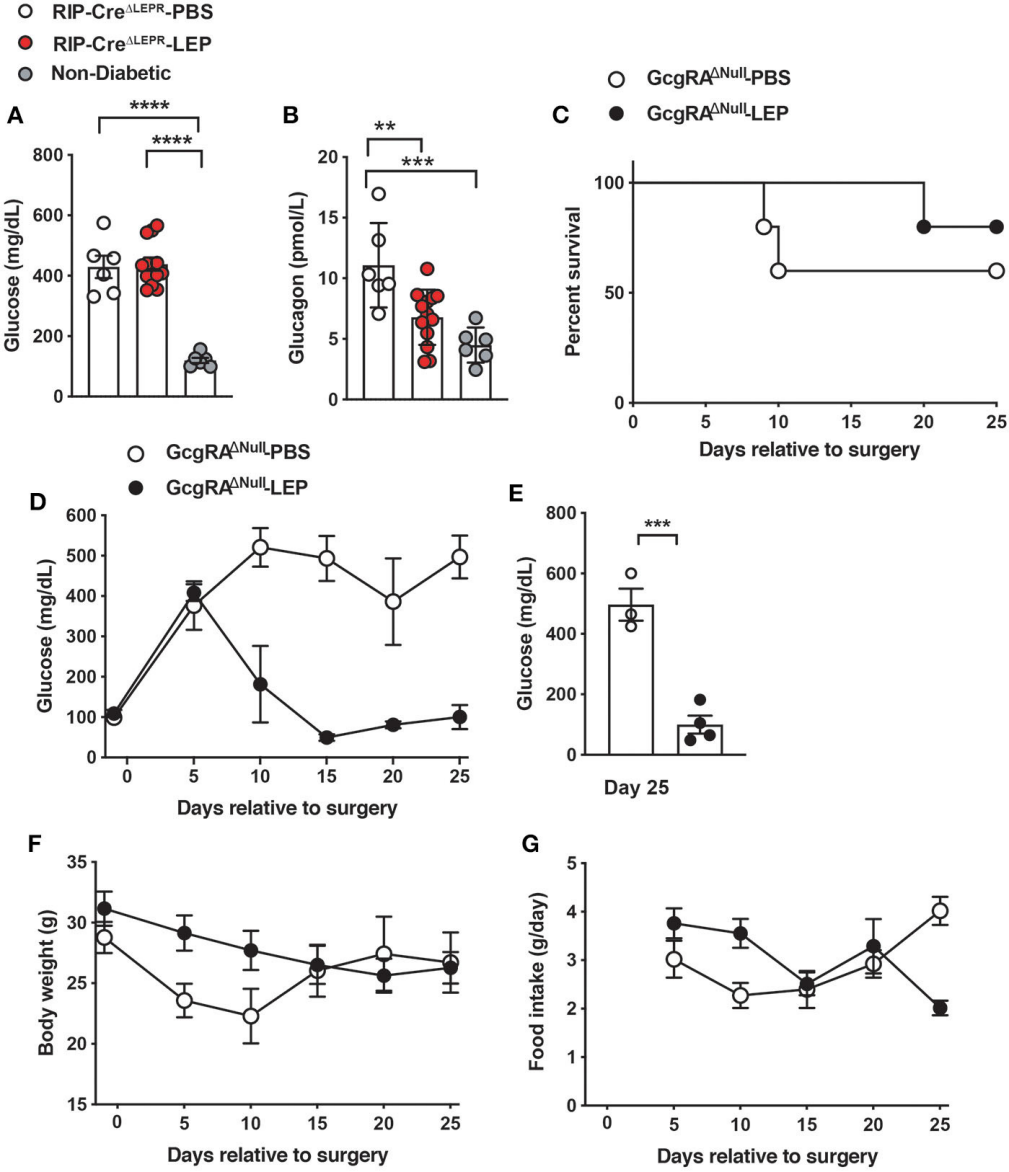
LEPRs in RIP-Cre^25Mgn^ neurons do not contribute leptin-induced suppression of glucagon secretion in insulin-deficient mice. **(A)** Blood glucose (*n* = 6; RIP-Cre^ΔLEPR^-PBS, 12; RIP-Cre^ΔLEPR^-LEP, and 6; Non-diabetic) and **(B)** plasma glucagon levels (*n* = 6; RIP-Cre^ΔLEPR^-PBS, 13; RIP-Cre^ΔLEPR^-LEP, and 6; Non-diabetic) in insulin-deficient RIP-Cre^ΔLEPR^ mice 10 days after the induction of chronic administration of leptin into the lateral ventricle (25 ng/0.11 μL/h). Control group for leptin administration was administered sterile vehicle (PBS). Non-diabetic group was composed of littermate *Lepr*^flox/flox^ mice that were not administered either i.p. DT or i.c.v. leptin/PBS. **(C)** The survival percentage (*n* = 5; GcgRA^ΔNull^-PBS and 5; GcgRA^ΔNull^-LEP at day −1), **(D)** the time course of blood glucose (*n* = 5; GcgRA^ΔNull^-PBS and 5; GcgRA^ΔNull^-LEP at day −1), **(E)** blood glucose at Days 25 (*n* = 3; GcgRA^ΔNull^-PBS and 4; GcgRA^ΔNull^-LEP), **(F)** the time course of body weight (*n* = 5; GcgRA^ΔNull^-PBS and 5; GcgRA^ΔNull^-LEP at day −1) **(G)** the time course of food intake (*n* = 5; GcgRA^ΔNull^-PBS and 5; GcgRA^ΔNull^-LEP at day 5) in insulin-deficient *Gcg* knockout mice. Values are mean ± S.E.M. ****p < 0.0001, ***p < 0.001, **p <0.01. One way ANOVA followed by Tukey's multiple comparison test **(A,B)**, Log-rank (Mantel-Cox) testing **(C)**, unpaired *t*-test **(E)** were used to analyze the data.

Glucagon is one of the key factors contributing to hyperglycemia in insulin deficiency (42–44). Leptin injection can lower hyperglucagonemia in insulin-deficient rodents (1, 41, 45), suggesting that suppression of hyperglucagonemia is key for glucose-lowering effects of leptin. We examined blood glucagon levels in RIP-Cre^ΔLEPR^-LEP, however, i.c.v. leptin injection lowered blood glucagon in insulin-deficient RIP-Cre^ΔLEPR^ mice (Figure 4B), while RIP-Cre^ΔLEPR^-LEP still showed hyperglycemia. These data suggest that hyperglucagonemia is not a driving-factor for hyperglycemia seen in RIP-Cre^ΔLEPR^-LEP. Intriguingly, blood glucagon levels in RIP-Cre^RA−LEPR^ mice were higher than that of WT-LEP (Supplementary Figure 2A), suggesting that LEPRs in RIP-Cre are neither required nor sufficient to restore normal glucagonemia. This result brought the question of what a role of glucagon for glucose-lowering effects of leptin is. Intriguingly, previous studies have indicated that glucose-lowering effects of leptin are not necessarily correlated with circulating glucagon levels (46–49). We further determined if glucose-lowering effects of leptin can be executed independently of glucagon signaling. To do so, we utilized mice lacking the preproglucagon gene, *Gcg* (GcgRA^ΔNull^) (27). We chronically i.c.v. administered leptin into insulin-deficient GcgRA^ΔNull^ mice (GcgRA^ΔNull^-LEP) and examined their blood glucose levels, survival rate, body weight, and food intake. I.c.v. PBS injection did not reverse hyperglycemia in insulin-deficient GcgRA^ΔNull^ mice (GcgRA^ΔNull^-PBS) (Figure 4D). Interestingly, i.c.v. leptin administration normalized blood glucose levels in insulin-deficient GcgRA^ΔNull^ mice (Figures 4D,E).

Of note, the survival rate of Gcg^KO^-PBS was ~60% at 25 days after induction of insulin deficiency (Figure 4C). All our previous studies have shown that insulin deficiency caused by the RIP^Herr^-DTR method leads to decease in mice within 2–3 weeks (6, 8, 26). These facts suggest that hyperglucagonemia has a negative impact on the survivability of insulin-deficient mice. We speculated that restoration of glucagon levels by i.c.v. leptin administration (Figure 4B) may contribute to the improvements of survival rate in RIP-Cre^ΔLEPR^-LEP (Figure 2). Further studies will be warranted to investigate the role of glucagon in leptin-induced reverse effects of lethality. Collectively, these data indicate that glucose-lowering effects of leptin in an insulin-independent manner do not rely on glucagon system, and maybe other peptides derived from preproglucagon such as glucagon-like peptide-1 either.

### LEPRs in RIP-Cre^25Mgn^ Neurons Mediate Anti-dyslipidemia Effects of Leptin in an Insulin-Independent Manner

**Figure 5:**
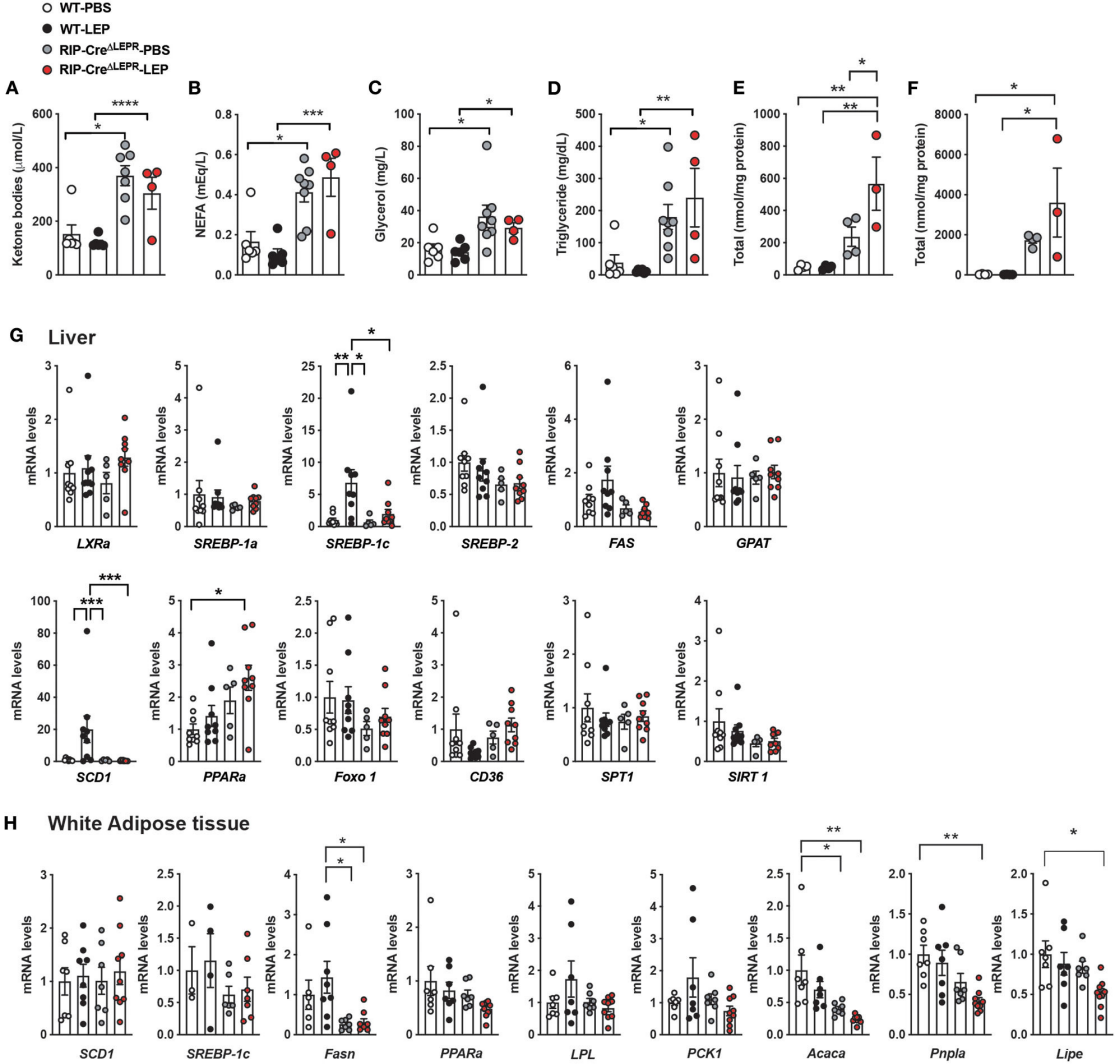
LEPRs in RIP-Cre^25Mgn^ neurons mediate anti-dyslipidemia effects of leptin in insulin-deficient mice. **(A)** Plasma ketone bodies (*n* = 6; WT-PBS, 6; WT-LEP, 4; RIP-Cre^ΔLEPR^-PBS, and 7; RIP-Cre^ΔLEPR^-LEP), **(B)** non-esterified fatty acids (NEFA) (*n* = 6; WT-PBS, 6; WT-LEP, 4; RIP-Cre^ΔLEPR^-PBS, and 8; RIP-Cre^ΔLEPR^-LEP), **(C)** Glycerol (*n* = 6; WT-PBS, 6; WT-LEP, 4; RIP-Cre^ΔLEPR^-PBS, and 8; RIP-Cre^ΔLEPR^-LEP), **(D)** TG (*n* = 6; WT-PBS, 6; WT-LEP, 4; RIP-Cre^ΔLEPR^-PBS, and 8; RIP-Cre^ΔLEPR^-LEP), **(E)** hepatic total FFAs level (*n* = 4; WT-PBS, 4; WT-LEP, 4; RIP-Cre^ΔLEPR^-PBS, and 3; RIP-Cre^ΔLEPR^-LEP), **(F)** hepatic total TG levels (*n* = 4; WT-PBS, 4; WT-LEP, 4; RIP-Cre^ΔLEPR^-PBS, and 3; RIP-Cre^ΔLEPR^-LEP) of insulin-deficient RIP-Cre^ΔLEPR^ mice administered leptin into the lateral ventricle (25 ng/0.11 μL/h). Blood and tissue samples were collected 10 days after the induction of chronic administration of leptin into the lateral ventricle. mRNA levels of genes related to lipid metabolism in **(G)** liver (*n* = 9; WT-PBS, 9; WT-LEP, 5; RIP-Cre^ΔLEPR^-PBS, and 9; RIP-Cre^ΔLEPR^-LEP) and **(H)** perigonadal white adipose tissue (*n* = 4–7; WT-PBS, 4–8; WT-LEP, 6–7; RIP-Cre^ΔLEPR^-PBS, and 6–9; RIP-Cre^ΔLEPR^-LEP). Control groups were administered sterile PBS. Values are mean ± S.E.M. ****p < 0.0001, ***p < 0.001, **p <0.01, *p < 0.05. One way ANOVA followed by Tukey's multiple comparison test was used to analyze the data.

Leptin can improve dyslipidemia in insulin-deficient rodents (1, 41, 47). Recent studies have pinpointed that improvements of aberrant fat metabolism contribute to glucose-lowering effects of leptin in insulin-deficient rodents (47, 50, 51). For instance, administration of fatty acid emulsion into bloodstream (47) or i.p. glycerol injection (51) can reverse glucose-lowering effects of leptin in insulin-deficient rodents. Excess circulating lipids disrupt hepatic glucose metabolism, leading to excess hepatic glucose production (50). To determine whether dyslipidemia could contribute to preventing leptin for glucose-lowering effects in insulin-deficient RIP-Cre^ΔLEPR^ mice, we first measured circulating FFAs, ketone bodies, glycerol, and TG in RIP-Cre^ΔLEPR^-LEP at 10 days after the beginning of leptin administration. Intriguingly, RIP-Cre^ΔLEPR^-LEP and RIP-Cre^ΔLEPR^-PBS showed significantly higher levels of all of these fat substrates (Figures 5A–D). We further examined hepatic FFAs and TG levels. Hepatic FFAs and TG levels in RIP-Cre^ΔLEPR^-LEP and RIP-Cre^ΔLEPR^-PBS were extremely higher compared to WT-LEP and WT-PBS (Figures 5E,F). Interestingly, blood FFAs levels in RIP-Cre^RA−LEPR^-LEP mice was significantly higher than WT-LEP (Supplementary Figure 2B), suggesting LEPRs in RIP-Cre^25Mgn^ neurons are not sufficient to mediate lipid-lowering effects of leptin. Of note, we confirmed again that RIP-Cre^ΔLEPR^-LEP showed hyperglycemia while body weight and food intake between RIP-Cre^ΔLEPR^-LEP and WT-LEP were comparable (Supplementary Figures 3A–C).

To determine if hepatic lipid synthesis is dysregulated that leads to the high levels of FFAs and TG in RIP-Cre^ΔLEPR^-LEP and RIP-Cre^ΔLEPR^-PBS, we measured mRNA levels of genes related to lipid synthesis and oxidation. As previously reported (41), leptin can restore mRNA levels of *Srebp-1c* and *Scd-1* in liver (Figure 5G) in an insulin-independent manner. Interestingly, i.c.v. leptin administration did not restore mRNA levels of *Srebp-1c* and *Scd-1* in liver of insulin-deficient RIP-Cre^ΔLEPR^ mice (Figure 5G). We assume that excess hepatic lipids suppress genes related to *de novo* lipid synthesis (52) in RIP-Cre^ΔLEPR^-LEP and RIP-Cre^ΔLEPR^-PBS. These data indicate that liver unlikely generated an excess amount of *de novo* lipids in RIP-Cre^ΔLEPR^-LEP and RIP-Cre^ΔLEPR^-PBS, and we asked if lipid synthesis in adipose tissues would increase by assessing mRNA of genes related to lipogenesis and lipolysis. Central leptin signaling suppresses lipogenesis in white adipose tissues (WAT) of mice in the insulin-clamped condition (53). Because insulin deficiency induces the drastic reduction of WAT weight due to augmented lipolysis and decreased lipogenesis, we had difficulties in collecting WAT of mice in all groups at Days 10; therefore, we collected WAT at Days 5. Again, we observed that blood glucose levels in RIP-Cre^ΔLEPR^-LEP was extremely higher compared with WT-LEP at Days 5 (Supplementary Figure 3E). We did not find drastic increases in mRNA levels of gene related to lipogenesis and lipolysis in perigonadal WAT of RIP-Cre^ΔLEPR^-LEP and RIP-Cre^ΔLEPR^-PBS (Figure 5H). Rather, we found that mRNA levels of *Fasn, Pnpla*, and *Lipa* in RIP-Cre^ΔLEPR^-LEP were significantly lower compared to WT-PBS, suggesting that the excess blood lipids did not resulted from *de novo* synthesis of lipid or lipolysis. Excess lipids unlikely result from dietary lipids, because the amount of food intake in RIP-Cre^ΔLEPR^-LEP was comparable of that in WT-LEP at 5 days and 10 days (Figure 2 and Supplementary Figure 3C), and we used normal a chow diet that contains relatively low fat (17 % of total calories). Further studies will be warranted to identify organs/cells that generate excess circulating lipids in insulin-deficient RIP-Cre^ΔLEPR^ mice.

**Figure 6:**
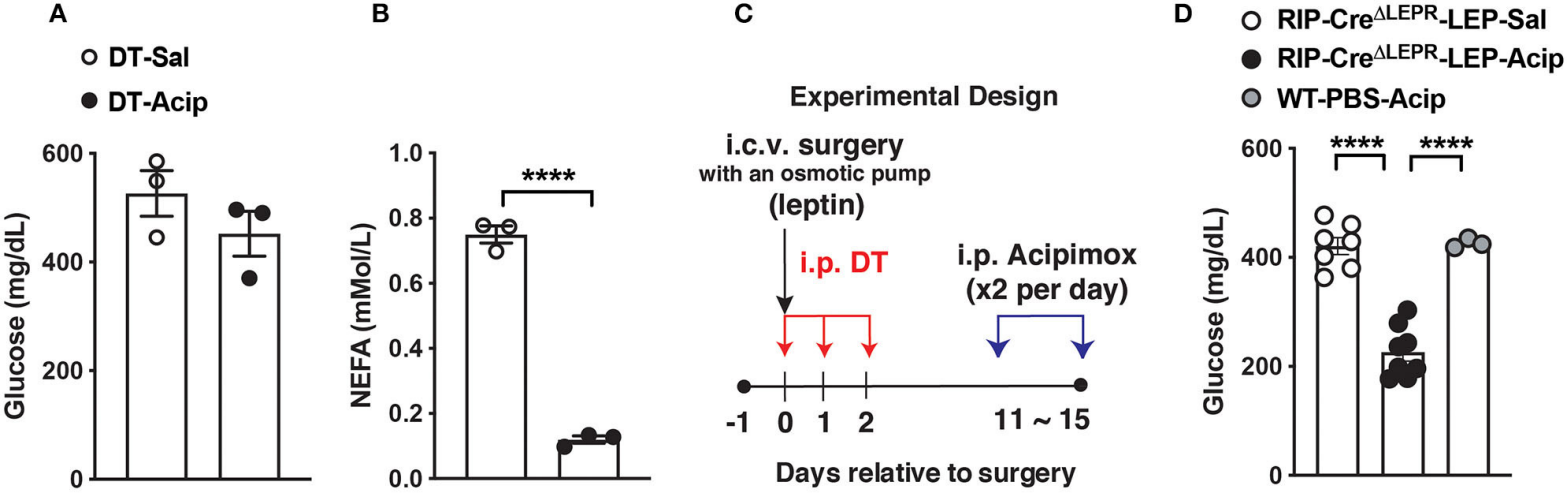
Lowering blood lipids levels reverses hyperglycemia in insulin-deficient RIP-Cre^ΔLEPR^ mice administered leptin. **(A)** Blood glucose and **(B)** plasma NEFA (*n* = 3; WT-Sal, and 3; WT-Acp) levels in insulin-deficient mice administered Acipimox (WT-Acip) or vehicle (Saline, WT-Sal). RIP-DTR mice were administered DT as described in Figure 1 to induce insulin deficiency. **(C)** Experimental design for D. **(D)** Blood glucose level 5 days after i.p. acipimox injection (2 times per day for 5 days at the dose of 100 mg/kg B.W.) into insulin-deficient RIP-Cre^ΔLEPR^ mice chronically administered leptin into the lateral ventricle (25 ng/0.11 μL/h) (RIP-Cre^ΔLEPR^-LEP-Acip) (*n* = 7; RIP-Cre^ΔLEPR^-LEP-Sal, 8; RIP-Cre^ΔLEPR^-LEP-Acip, and 3; WT-PBS-Acip). Control group for i.p., acipimox injection was administered i.p., saline into insulin-deficient RIP-Cre^ΔLEPR^ mice chronically administered leptin into the lateral ventricle RIP-Cre^ΔLEPR^-LEP-Sal). Control group to determine effects of chronic acipimox injection itself on glucose levels in insulin-deficient mice was WT mice administered i.c.v. PBS and i.p., acipimox (WT-PBS-Acip). Values are mean ± S.E.M. ****p < 0.0001. Unpaired *t*-test **(A,B)** and one way ANOVA followed by Tukey's multiple comparison test **(D)** were used to analyze the data.

Finally, we asked if suppression of excess blood lipids by acipimox, which reduces lipids in blood (54), can reverse hyperglycemia in RIP-Cre^ΔLEPR^-LEP. An acipimox injection alone (i.p. 100 mg/kg BW, single injection) dramatically reduced blood FFAs but not glucose levels in RIP^Herr^-DTR-induced insulin-deficient mice (Figures 6A,B). I.p. administration acipimox into RIP-Cre^ΔLEPR^-LEP (two times per day for 5 days) 10 days after leptin administration initiated (Figure 6C) significantly improved hyperglycemia RIP-Cre^ΔLEPR^-LEP (RIP-Cre^ΔLEPR^-LEP-Acip) compared to the control group (RIP-Cre^ΔLEPR^-LEP-Sal) (Figure 6D), along with the improvements of blood FFAs levels (Supplementary Figure 4A). Collectively, our studies support that lipid-lowering actions are critical for glucose-lowering effects of leptin, and demonstrate that LEPRs in RIP-Cre^25Mgn^ neurons mediate anti-dyslipidemia actions of leptin in an insulin-independent manner.

## Discussion

In the present study, we identify that LEPRs in RIP-Cre^25Mgn^ neurons are required for lipid-lowering effects of leptin, thereby glucose-lowering effects in an insulin-independent manner. Our data suggest that glucagon signaling does not drive hyperglycemia in insulin-deficient mice lacking LEPRs in RIP-Cre^25Mgn^ administered i.c.v. leptin (Figure 4). Rather, our data indicate that excess circulating lipids contribute to the refractory responses of insulin-deficient RIP-Cre^ΔLEPR^ mice to glucose-lowering effects of leptin in the absence of insulin (Figures 5, 6). Collectively, we propose that LEPRs in RIP-Cre^25Mgn^ neurons are key to regulate lipid metabolism in an insulin-independent manner, although target peripheral tissues have to be determined in future studies.

Our approaches in this study could not allow us to decipher the precise anatomical location of RIP-Cre^25Mgn^ neurons contributing to the regulation of lipid metabolism because of the broad expression pattern of Cre recombinase in RIP-Cre^25Mgn^ mice (12, 13) and that LEPRs are also expressed broadly throughout the hypothalamus (31, 55). Nonetheless, we assume that GABAergic RIP-Cre^25Mgn^ neurons in the ARC and/or DMH are key for anti-dyslipidemia actions of leptin in insulin-deficient mice, because (i) leptin-responsive GABAergic neurons are located only in the ARC, DMH, and LHA, and (ii) GABAergic RIP-Cre^25Mgn^ neurons are anatomically limited to the ARC, DMH, and MTu; therefore, ARC and DHM are only overlapped regions that match to the anatomical and chemical-classification profiling from previous studies. Although studies has shown that GABAergic ARC (e.g., AgRP neurons) (56–59) and DMH (e.g., LEPRs-neurons) (60) neurons regulate multiple aspects of metabolism including food intake and energy expenditure, the role of these neurons in the regulation of lipid metabolism, in particular in an insulin-independent manner, remains unclear.

RIP-Cre^25Mgn^ neurons in the ARC (ARC RIP-Cre^25Mgn^ neurons) are distinct from AgRP neurons (10), which also contribute to glucose-lowering effects of leptin in an insulin-independent manner (7, 8). ARC RIP-Cre^25Mgn^ neurons are composed of at least 10 genetically distinguished neuronal groups (10). Within these groups, 8 neuronal clusters are categorized into GABAergic neurons (*Nfix*/*Ht2c*-, *Arx*/*Nr5a2*-, *Th*/*Slc6a3*-, *Th*/*Nfib*-, *Sst*/*Unc13c*-, *SSt*/*Pthlh*-, *Htr3b*-, and *Tbx19*-expressing neurons), and none of them are AgRP neurons (10). A previous study shows that leptin acts on ARC GABAergic RIP-Cre^25Mgn^ neurons to increase energy expenditure along with thermogenesis in interscapular brown adipose tissue without affecting food intake behavior (17). Mice lacking LEPRs in RIP-Cre^25Mgn^ neurons exhibit lower energy expenditure without changing food intake compared to control mice (21). Compared to ARC RIP-Cre^25Mgn^ neurons, the genetic property of RIP-Cre^25Mgn^ neurons in the DMH (DMH RIP-Cre^25Mgn^ neurons) at the single cell level is still undetermined. In addition, the role of DMH RIP-Cre^25Mgn^ neurons in the regulation of metabolism is completely unknown. Further studies will be warranted to pinpoint the specific neuronal group(s) within RIP-Cre^25Mgn^ neurons that regulate fat metabolism in an insulin-independent manner.

Glucose-lowering effects of leptin in insulin-deficient rodents are abolished by lipids infusion (47). Perry and her colleagues propose that anti-dyslipidemia actions of leptin are mediated by the HPA-axis because the infusion of corticosterone reverses leptin-induced improvements on aberrant blood FFAs and ketone bodies as well as hyperglycemia in streptozocin (STZ)-administered diabetic rats (47). Contrary to their finding, Morton and his colleagues argue that the HPA-axis does not contribute to glucose-lowering effects of leptin because (i) STZ administrations cause diabetes in adrenalectomized rats, and (ii) corticosterone administration in adrenalectomized rats does not reverse the glucose-lowering effects of leptin (61). Additionally, a study using hypophysectomized Sprague-Dawley rats demonstrates that the pituitary gland is not required for glucose-lowering effects of leptin (62), suggesting that the HPA-axis may not be the primary factor to mediate the effects. Of note, the experimental conditions may contribute to the discrepancy between them as Perry and colleagues performed experiments in the short-term (within hours) (47), while others conducted in the long-term (over days) (61, 62). We found that plasma corticosterone levels in RIP-Cre^ΔLEPR^-LEP tended to be higher than WT-LEP (Supplementary Figure 3D). Surprisingly, plasma corticosterone levels in RIP-Cre^ΔLEPR^-LEP-Acip was significantly higher that RIP-Cre^ΔLEPR^-LEP-Sal (Supplementary Figure 4B), although acipimox injection improved glucose and FFAs in RIP-Cre^ΔLEPR^-LEP (Figure 6 and Supplementary Figure 4A). This result is puzzling, nonetheless, these data further support that lowering corticosterone may not be required for leptin to exert its glucose-lowering effects in insulin-deficient mice in the long-term. Further studies are warranted the mechanism by which LEPRs RIP-Cre^25Mgn^ neurons regulates lipid metabolism in an insulin-independent manner.

The CNS-peripheral pathway underlying hypothalamic regulation of lipid metabolism by leptin in the absence of insulin remains largely unclear. Decreased leptin levels by fasting trigger lipolysis in WAT via activation of HPA-axis, leading to glucose-counterregulatory actions in response to starvation-induced hypoglycemia (63). The sympathetic nervous system (SNS) also mediates lipogenesis and lipolysis in WAT by hypothalamic leptin signaling (53). Of note, most, if not all, of these aforementioned studies have been conducted in the presence of insulin such as normal metabolic rodents or type 2 diabetic rodents (64, 65). Glucose-lowering effects of leptin with and without insulin are mediated by different neuronal populations (66), thereby mechanisms of hypothalamic regulation of lipid metabolism by leptin may differ between in the absence and presence of insulin. As we mentioned above, HPA-axis and the SNS contribute to hypothalamic regulation of lipid metabolism by leptin in the presence of insulin. Studies have shown that leptin can restore normal glycemia in the absence of insulin while HPA-axis or the SNS is removed from the system (61, 62, 67, 68), implicating that they are not required for lipid-lowering effects of leptin in an insulin-independent manner. Further studies are needed to decipher the factor which connects the hypothalamus to peripheral tissues to mediate lipid-lowering effects of leptin in an insulin-independent manner.

In summary, our current study demonstrates that LEPRs in RIP-Cre^25Mgn^ neurons significantly contribute to glucose-lowering effects of leptin in an insulin-independent manner by reversing aberrant lipid metabolism. It is still unclear whether LEPRs in RIP-Cre^25Mgn^ neurons mediate effects of leptin on lipid metabolism such as increases of fatty acid oxidation and lipolysis in adipose tissues in the presence of insulin. Unraveling the mechanism by which LEPRs in RIP-Cre^25Mgn^ neurons regulate lipid metabolism may pave a way to design new treatments for several forms of diabetes.

## Data Availability Statement

The raw data supporting the conclusions of this article will be made available by the authors, without undue reservation.

## Ethics Statement

The animal study was reviewed and approved by The Institutional Animal Care and Use Committee of the University of Texas Southwestern Medical Center and the Institutional Animal Care and Use Committee of the University of Texas Health San Antonio. Written informed consent was obtained from the owners for the participation of their animals in this study.

## Author Contributions

AS performed and analyzed experiments and edited the manuscript. JP designed, performed, and analyzed experiments, and edited the manuscript. MP performed and analyze experiments. SF performed experiments. DS generated *Gcg*^loxTB/WT^ mice and edited manuscript. XH supervised experiments and edited manuscript. TF designed, performed, supervised, analyzed experiments, and wrote and finalized the manuscript. All authors contributed to the article and approved the submitted version.

## Conflict of Interest

The authors declare that the research was conducted in the absence of any commercial or financial relationships that could be construed as a potential conflict of interest.
