# Supplementary material for: Leptin Receptors in RIP-Cre25Mgn Neurons Mediate Anti-dyslipidemia Effects of Leptin in Insulin-Deficient Mice
Source: Front Endocrinol (Lausanne). 2020 Sep 23;11:588447. doi: 10.3389/fendo.2020.588447 (PMC7538546; doi:10.3389/fendo.2020.588447)

Supplemental Figure 1: DT injections ablate pancreatic β-cells of mice lacking LEPRs in RIP-Cre^{25Mgn} neurons. (A) Insulin levels in the plasma of insulin-deficient mice induced by a RIP^{Herr}-DTR approach. These mice were described in Figure 1, 2 and 4. Non-diabetic RIP-DTR mice were injected with sterile PBS. (**B**) Blood glucose and (**C**) plasma insulin levels in mice lacking LEPRs in RIP-Cre^{25Mgn} neurons (RIP-Cre$^{\Delta LEPR}$). DT was administered as described in the Method section (0.5 μg/kg B.W. per day, total 3 injections). n = 3-10. Values are mean ± S.E.M. **** $p < 0.0001$. Unpaired t-test was used to analyze the data.

Supplemental Figure 2. Related to Figure 3. Re-expression of LEPRs in RIP-Cre^{25Mgn} neurons is not sufficient to reserve dyslipidemia in insulin deficiency by i.c.v. leptin administration. (A) Plasma glucagon, (**B**) NEFA, and (**C**) corticosterone in mice re-expressing LEPRs only in RIP-Cre^{25Mgn} neurons (RIP-Cre$^{RA\text{-}LEPR}$). Wild-type control (WT) was composed of RIP-Cre^{25Mgn}::*Lepr*$^{WT/WT}$ and *Lepr*$^{WT/WT}$, and *Lepr*$^{loxTB/loxTB}$ mice were used as LEPRs-deficient mice. n = 4-6. Values are mean ± S.E.M. ** $p < 0.01$. Unpaired t-test was used to analyze the data.

Supplemental Figure 3. Related to Figure 5. Metabolic parameters of mice in Figure 5G and H (A) The time course of blood glucose levels, (**B**) body weight, and (**C**) food intake in insulin-deficient RIP-Cre$^{\Delta LEPR}$ mice chronically administered leptin into the lateral ventricle (25 ng/0.11 μL/hour) (n = 10; WT-PBS, 10; WT-LEP, 12; RIP-Cre$^{\Delta LEPR}$-PBS, and 13; RIP-Cre$^{\Delta LEPR}$-LEP at day −1). Corticosterone levels in insulin-deficient RIP-Cre$^{\Delta LEPR}$ mice 10 days after the induction of chronic administration of leptin into the lateral ventricle (25 ng/0.11 μL/hour) (n = 7; WT-PBS, 6; WT-LEP, 8; RIP-Cre$^{\Delta LEPR}$-PBS, and 8; RIP-Cre$^{\Delta LEPR}$-LEP). (**E**) Blood glucose and (**F**) body weight in insulin-deficient RIP-Cre$^{\Delta LEPR}$ mice chronically administered leptin into the lateral ventricle (25 ng/0.11 μL/hour) (n = 7; WT-PBS, 8; WT-LEP, 7; RIP-Cre$^{\Delta LEPR}$-PBS, and 9; RIP-Cre$^{\Delta LEPR}$-LEP at day −1). Insulin deficiency was induced by administration of DT. Control group for leptin

administration was administered sterile vehicle (PBS). Genetic control group (WT) did not bear RIP-Cre^{25Mgn}. Values are mean ± S.E.M. One way ANOVA followed by Tukey's multiple comparison test (D) was used to analyze the data.

Supplemental Figure 4. Related to Figure 6. Lowering blood lipids levels increases blood corticosterone levels in insulin-deficient RIP-Cre$^{\Delta LEPR}$ mice administered leptin. (**A**) Plasma NEFA, (**B**) glucagon, and (**C**) corticosterone levels 5 days after i.p. acipimox injection (2 times per day for 5 days at the dose of 100 mg/kg B.W.) into insulin-deficient RIP-Cre$^{\Delta LEPR}$ mice chronically administered leptin into the lateral ventricle (25 ng/0.11 μL/hour) (RIP-Cre$^{\Delta LEPR}$-LEP-Acip) (n = 7; RIP-Cre$^{\Delta LEPR}$-LEP-Sal, 8; RIP-Cre$^{\Delta LEPR}$-LEP-Acip). Control group was administered i.p. saline into insulin-deficient RIP-Cre$^{\Delta LEPR}$ mice chronically administered leptin into the lateral ventricle (RIP-Cre$^{\Delta LEPR}$-LEP-Sal). Values are mean ± S.E.M. * $p < 0.05$, *** $p < 0.001$. Unpaired t-test was used to analyze the data.

Supplemental Table 1. Sequences of genotyping primers

Supplemental Table 2. Sequences of qPCR primers

Supplemental Figure 1

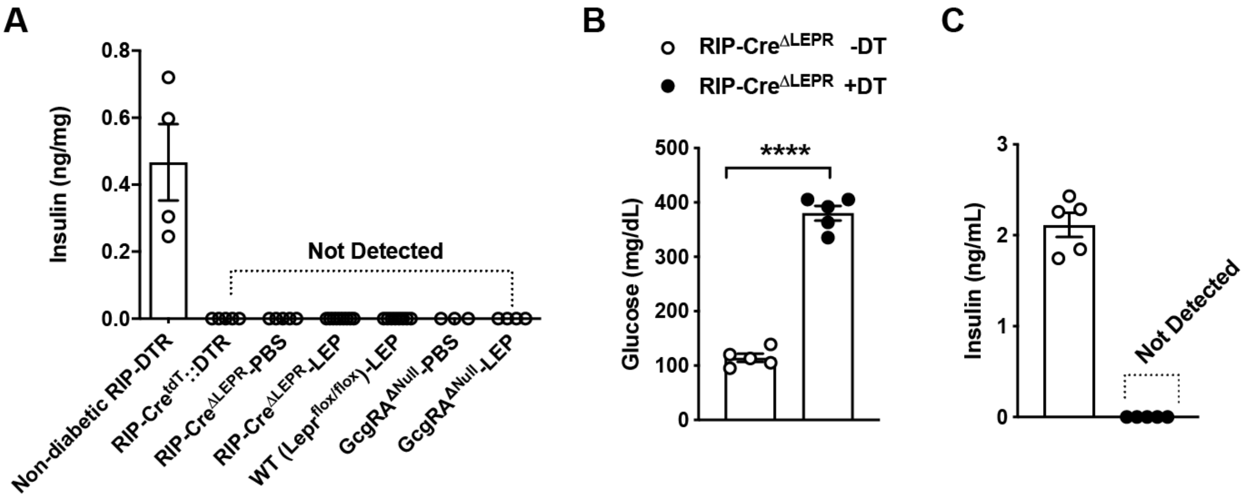

Supplemental Figure 2

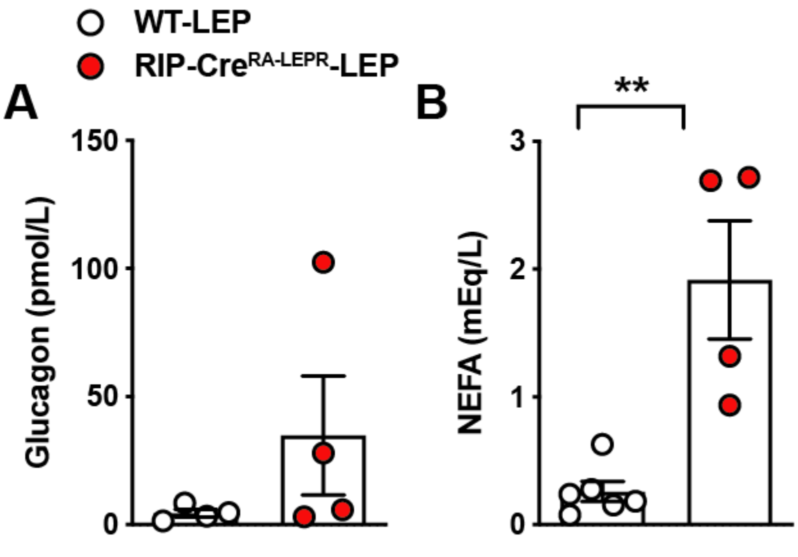

Supplemental Figure 3

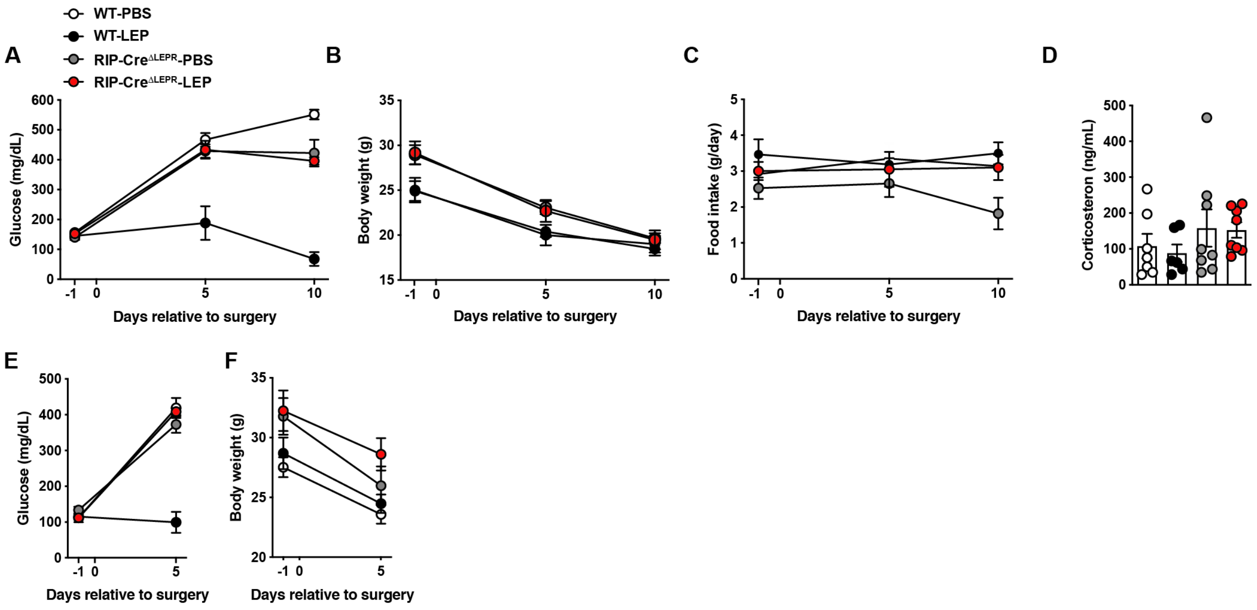

Supplemental Figure 4

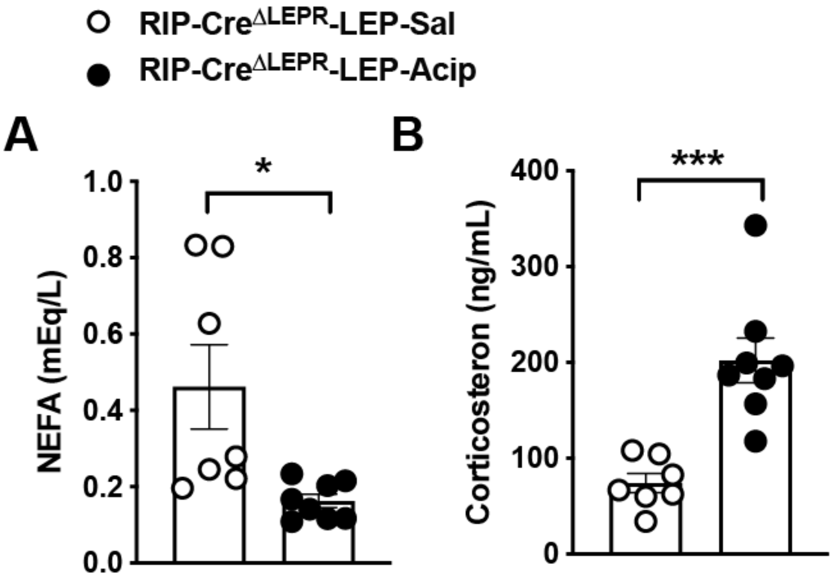

Supplement: Supplementary file 3 [file Data_Sheet_1.PDF]
